# Guillain-Barre syndrome in 220 patients with COVID-19

**DOI:** 10.1186/s41983-021-00310-7

**Published:** 2021-05-04

**Authors:** Josef Finsterer, Fulvio A. Scorza

**Affiliations:** 1 Klinik Landstrasse, Messerli Institute, Postfach 20, 1180 Vienna, Austria; 2 Disciplina de Neurociência. Universidade Federal de São Paulo/Escola Paulista de Medicina (UNIFESP/EPM), São Paulo, Brazil

**Keywords:** SARS-CoV-2, COVID-19, Guillain-Barre syndrome, Nerve conduction, Immunoglobulins

## Abstract

This review summarises and discusses recent findings concerning the pathophysiology, clinical presentation, diagnosis, treatment, and outcome of SARS-CoV-2-associated Guillain-Barre syndrome (SC2-GBS). By the end of December 2020, at least 220 patients with SC2-GBS have been published in 95 papers. SC2-GBS is most likely secondary due to an immune reaction against SARS-CoV-2 since the virus has not been found in the CSF of any SC2-GBS patient so far reported. SC2-GBS occurs in each age group and does not differ from non-SC2-GBS regarding clinical presentation and treatment, but the outcome of SC2-GBS is worse compared to non-CS2-GBS patients, and the prevalence/incidence of GBS most likely increased since the outbreak of the pandemic. Early diagnosis of SC2-GBS is warranted to apply appropriate treatment in due time and to improve the overall outcome from the infection.

## Introduction

Since the outbreak of the pandemic by the SARS-CoV-2 virus, it became rapidly obvious that the virus not only causes lung disease (COVID-19) but affects other organs as well, particularly the central and peripheral nervous system (PNS, CNS), the kidneys, the intestines, and the heart [1–3]. The most disabling PNS disorder is polyradiculitis (polyradiculoneuritis, Guillain-Barre syndrome (GBS)) [4]. GBS comprises a number of subtypes which include acute, inflammatory, demyelinating neuropathy (AIDP) (classic type), acute, motor, axonal neuropathy (AMAN), acute, motor and sensory, axonal neuropathy (AMSAN), Miller-Fisher syndrome (MFS), polyneuritis cranialis (PNC), the pharyngeal, cervical, and brachial (PCB) variant, and Bickerstaff encephalitis (BFE) [5]. GBS is usually diagnosed according to the Brighton criteria if there is bilateral, progressive, flaccid lower > upper limb paraparesis, if tendon reflexes in weak limbs are diminished, if the disease course is monophasic and if time between onset and nadir ranges from 12 h to 28 days, if cerebrospinal fluid (CSF) investigations reveal a cell count < 50cells/μL, if CSF protein is elevated (dissociation cyto-albuminque (DCA)), and if nerve conduction studies show a demyelinating lesion of motor nerves (AIDP), an axonal lesion of motor nerves (AMAN), or an axonal lesion of motor and sensory nerves (AMSAN) [5]. MFS is diagnosed if there is acute onset ophthalmoplegia, areflexia, ataxia, and DCA. PNC is diagnosed in case of a lesion of a single or multiple cranial nerves and DCA. PCB is diagnosed if there is progressive dysphagia, dysphonia, upper limb weakness, and DCA [5]. BFE is diagnosed if there are pyramidal signs and impaired consciousness in addition to MFS. In the early stages of GBS, upper or lower limb paraplegia with preserved tendon reflexes may occur [5]. There can be even hyperreflexia if the pyramidal tract is involved. All GBS subtypes occur in the setting of a preceding viral or bacterial infection. The type of preceding infection largely determines the subtype and clinical course of GBS. This systematised review summarises and discusses recent findings and future perspectives concerning the pathophysiology, clinical presentation, diagnosis, treatment, and outcome of SARS-CoV-2-associated GBS (SC2-GBS).

## Methods

**Fig. 1:**
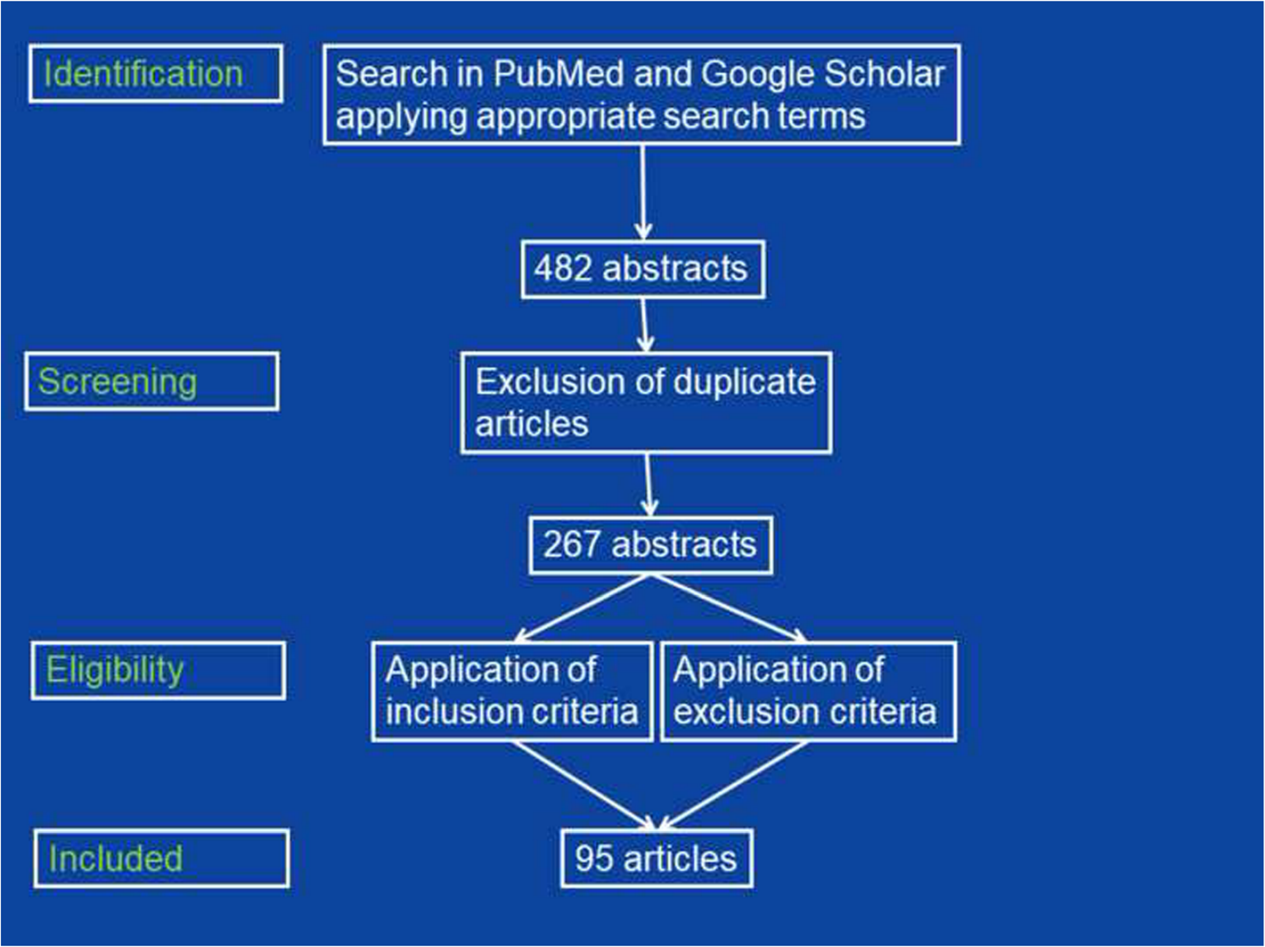
Flow chart detailing the search protocol and the results after application of inclusion and exclusion criteria

A systematised literature search in the databases PubMed and Google Scholar using the search terms “neuropathy,” “Guillain Barre syndrome,” “polyradiculitis,” “AIDP,” “AMAN,” “AMSAN,” “Miller-Fisher syndrome,” “polyneuritis cranialis,” and “Bickerstaff encephlaitis,” in combination with “SARS-CoV-2,” “COVID-19,” and “coronavirus” was conducted. Additionally, reference lists were checked for further articles meeting the search criteria. Included were only original articles detailing individual patients’ data (age, sex, latency between onset of COVID-19 and SC2-GBS, GBS subtype, results of CSF investigations, treatment, and outcome) and written in English, French, Spanish, Italian, or German, Excluded from data analysis were reviews, abstracts, proceedings, and editorials as well as original studies not specifying individual patients’ data (Fig. 1).

## Main text

By the end of December 2020, at least 220 patients with SC2-GBS have been published in 95 papers (Tables 1 and 2). Age of these patients (reported in *n* = 215) ranged from 8–94 years (Table 1). Gender (reported in *n* = 213) was male in 146 and female in 67 (Tables 1 and 2). Onset (reported in *n* = 165) was identified after/together with/before onset of non-neurological COVID-19 manifestations in 156/3/6 patients (Tables 1 and 2). Latency between onset of COVID-19 and GBS (*n* = 194) ranged from − 10 to 90 days. One patient remained asymptomatic. The GBS subtype (reported in *n* = 152) was identified as AIDP (*n* = 118), AMAN (*n* = 13), AMSAN (*n* = 11), MFS (*n* = 7), PNC (*n* = 2), the PCB variant (*n* = 1), and BFE (*n* = 0). SARS-CoV-2 was not detected in the CSF in any of the patients (Table 1). Therapy of GBS (reported in *n* = 215) comprised intravenous immunoglobulins (IVIG) (*n* = 191), plasmapheresis (*n* = 15), steroids (*n* = 2), or no therapy (*n* = 7) (Tables 1 and 2). Forty-one patients required artificial ventilation (Tables 1 and 2). Outcome (reported in *n* = 168) was assessed as complete recovery (*n* = 37), partial recovery (*n* = 119), or death (*n* = 12) (Tables 1 and 2). No studies about factors determining the outcome of SC2-GBS subtypes were identified.

**Table 1:** Patients with SARS-CoV-2 associated polyradiculitis as reported by the end of December 2020

Age (years)	Sex	Onset	LOO (days)	Subtype	CIC	CM	IVIG	AV	Recovery	Country
61	f	B	9	AIDP	nr	No	Yes	No	Yes	China
65	m	A	9	AMSAN	nd	DM	Yes	No	nr	Iran
54	m	A	8	AIDP	nr	No	Yes	Yes	Yes	USA
70	f	A	23	AIDP	nd	No	Yes	Yes	nr	Italy
66	f	A	7	AIDP	No	nr	Yes	Yes	Yes	Italy
54	f	A	21	AIDP	nd	No	Yes	No	Yes	Germany
70	f	A	3	AMSAN	No	RA	Yes	No	Partial	Morocco
20	m	A	5	AMAN	nd	No	Yes	No	Yes	India
71	m	A	4	AIDP	No	AHT, AAR, LC	Yes	Yes	Death	Italy
64	m	A	11	AIDP	nd	No	Yes	Yes	nr	France
nr	nr	A	7	AIDP	No	nr	Yes	No	Partial	Italy
nr	nr	A	10	AIDP	No	nr	Yes	No	Yes	Italy
nr	nr	A	10	AMAN	No	nr	Yes	Yes	Partial	Italy
nr	nr	A	5	AMAN	No	nr	Yes	No	Partial	Italy
nr	nr	A	7	AMAN	No	nr	Yes, PE	No	nr	Italy
50	m	A	3	MFS, PNC	No	No	Yes	No	Yes	Spain
39	m	A	3	MFS, PNC	No	No	No	No	Yes	Spain
61	m	A	10	MFS	No	No	S	No	Yes	Spain
76	f	A	8	GBS (no NCS)	nd	No	No	nr	Death	Spain
~ 75	m	B	10	AIDP	No	No	Yes	No	Yes	Swiss
43	m	A	10	AIDP	nr	nr	Yes	No	Yes	Spain
64	m	A	23	AIDP	No	nr	Yes	No	Yes	France
72	m	A	7	AIDP	No	AHT, CHD, AL	Yes	Yes	Partial	USA
~ 65	m	A	17	AIDP	No	No	Yes	No	Yes	Italy
67	f	A	10	nr	No	Breast cancer	PE	Yes	Partial	USA
54	m	A	14	AIDP	nd	nr	Yes	No	Partial	USA
43	m	A	21	AIDP	No	nr	Yes	No	Yes	France
71	f	A	10	AIDP	No	nr	Yes	No	Partial	France
36	m	A	4	MFS	nr	nr	Yes	No	Yes	USA
55	m	A	20	AIDP	No	nr	Yes	Yes	Partial	Italy
60	m	A	3	AMSAN	No	nr	Yes	Yes	Partial	Italy
58	m	AB	0	AIDP	No	No	Yes	No	Partial	Canada
52	f	A	15	AIDP	No	nr	Yes	No	Partial	Swiss
63	f	A	7	AIDP	nr	nr	Yes	No	Yes	Swiss
61	f	A	22	AIDP	No	nr	Yes	No	Partial	Swiss
53	f	B	nr	AIDP	No	No	PE	No	Partial	Turkey
51	f	A	14	MFS	nr	nr	Yes	No	Partial	Spain
56	f	A	15	AIDP	No	nr	Yes	Yes	Partial	Spain
68	m	A	14	AIDP	nr (ASPC)	nr	Yes, PE	Yes	Partial	Austria
55	f	A	14	AIDP	nr	No	Yes	Yes	Partial	Spain
53	m	A	24	AIDP	No	No	Yes	No	Yes	Netherlands
57	m	A	6	AIDP	No	AHT, psoriasis	Yes	Yes	Partial	UK
21	m	A	16	AIDP	nr	AHT, DM	PE	No	Yes	USA
41	m	A	10	AIDP	nr	DM	Yes	No	Partial	Iran
38	m	A	16	AIDP	nr	AHT	PE	No	Yes	Iran
14	f	A	nr	GBS	nr	No	Yes	No	Yes	Iran
49	m	A	14	AIDP	No	No	Yes	No	Yes	UK
68	m	A	5	AIDP	nr	AHT, HLP	Yes	No	Yes	Italy
11	m	A	21	AIDP	nr	No	Yes	No	Yes	Saudi
15	m	A	nr	AMAN	No	No	Yes	No	Partial	Brazil
72	m	A	18	AIDP	No	nr	Yes	Yes	Partial	Italy
72	m	A	30	AIDP	No	nr	Yes	Yes	Partial	Italy
49	f	A	14	AIDP	No	nr	Yes	No	Partial	Italy
94	m	A	33	AIDP	nr	nr	S	No	Partial	Italy
76	m	A	22	AIDP	No	nr	Yes	Yes	Partial	Italy
64	m	A	nr	GBS?	nr	DM	Yes	Yes	Yes	Japan
77	m	A	nr	AIDP	nr	AHT, HLP	Yes	No	Yes	Spain
58	f	A	6	AIDP	No	nr	PE	No	Yes	USA
56	f	A	7	AIDP	No	AHT, thyroxin ↓	nr	nr	Partial	Germany
61	f	A	14	AMAN	No	AHT, HLP	PE	No	Yes	USA
75	m	A	nr	nr	No	spinal trauma	Yes	No	Yes	USA
37	nr	A	10	nr	nr	nr	nr	nr	nr	Belgium
60	f	A	22	nr	nr	Migraine	Yes	No	Partial	USA
∅57	33 m	nr	0–37	nr	nr	nr	Yes, *n* = 46	nr	Death, *n* = 1	UK, *n* = 47
							PE, *n* = 1		nr, *n* = 46	
51	m	A	12	AIDP	No	nr	Yes	Yes	Partial	Germany
34	m	A	4	PNC	nr	Strabism	Yes	No	Partial	USA
71	f	A	Days	PNC	nr	AHT	No	No	Partial	USA
65	m	A	3	AIDP	nr	No	Yes	No	Yes	Germany
74	f	A	nr	AIDP	No	Lymphoma	Yes	No	Yes	Spain
49	m	A	14	MFS	No	Crohn’s disease	Yes	Yes	partial	USA
65	f	A	nr	AIDP	nr	Fibromyalgia	Yes	Yes	Death	Italy
12	m	A	7	nr	nr	No	Yes	Yes	Death	Tanzania
88	f	A	2	AMSAN	nr	nr	PE	Yes	Partial	Iran
47	m	A	7	AMSAN	nr	nr	PE	Yes	Death	Iran
58	m	A	9	AMSAN	nr	nr	Yes, PE	Yes	Death	Iran
54	m	A	3	nr	nr	GBS, DN	Yes	No	Yes	USA
57	m	A	nr	AMAN	nr	nr	Yes	No	nr	Italy
37	m	A	14	AIDP	nr	nr	Yes	Yes	Partial	Iran
41	m	A	10	AIDP	No	nr	Yes	No	Yes	Guinea
76	m	A	7	AIDP	No	Cardiomyopathy	Yes	No	Partial	France
∅59.2	22 m	A	16–35	AIDP, *n* = 23	nr	Several	*n* = 25	*n* = 5	Partial	UK, *n* = 30
				AMAN, *n* = 2			PE, *n* = 2			
44	m	A	nr	nr	nr	AHT, asthma	Yes	No	Yes	USA
54	f	A	20	AMAN	nr	Asthma	No	No	Partial	Japan
55	f	A	11	AMSAN	nr	Lung disease	Yes	Yes	Death	Iran
8	m	B	nr	AIDP	No	No	Yes	Yes	Partial	USA
65	m	A	14	AIDP	nr	nr	Yes	No	Partial	Iran
70	f	A	90	nr	nr	RSD	Yes	No	Yes	USA
55	f	A	10	AMAN	nr	DM, AHT	Yes	No	Partial	India
72	m	A	6	AIDP	nr	AHT	Yes	Yes	Death	India
55	m	A	7	AMSAN	nr	DM, AHT, RI	Yes	No	Partial	India
49	m	A	10	AIDP	nr	DM, AHT	Yes	No	Partial	India
53	m	A	nr	nr	nr	nr	Yes	No	Partial	Italy
36	m	A	18	AIDP	nr	AHT, NTX	Yes	Yes	Partial	USA
57	m	A	17	AIDP	nr	nr	Yes	No	Partial	Italy
∅53	11 m	A	0.5–28	AIDP	No, *n* = 4	nr	Yes, *n* = 15	nr	Partial,	Italy, *n* = 17
							PE, *n* = 2		Death, *n* = 1	
54	f	AB	0	nr	No	AHT	Yes	No	Partial	Spain
58	f	A	14	nr	nr	Disc prolapse	Yes	No	Partial	USA
65	m	A	nr	AIDP	nr	nr	Yes	No	Partial	Italy
73	m	AB	0	AIDP	No	nr	Yes	No	Partial	Italy
55	m	A	20	AIDP/MFS	No	nr	Yes	No	Partial	Italy
46	f	A	3	AIDP	No	nr	Yes	No	Partial	Italy
60	m	A	20	AMSAN	No	nr	Yes	No	Partial	Italy
63	f	A	15	AMSAN	nr	nr	Yes	No	Partial	Italy
~ 35	m	A	nr	AMAN	No	nr	Yes	No	Partial	UK
49	m	A	11	PCB	No	AHT, seminoma	No	No	Partial	Italy
54	m	A	4	AIDP	nr	AHT, obesity	Yes	Yes	Partial	Spain
54	nr	nr	nr	nr	No	AHT, HLP	Yes	Yes	Yes	Spain
72	f	A	8	AIDP	No	nr	Yes	Yes	Partial	Italy
48	m	A	18	AIDP	nr	DM	PE	No	Partial	USA
46	m	A	18	AIDP	nr	nr	No	No	Partial	Iran
65	m	A	10	AIDP	nr	nr	Yes	No	Partial	Iran
66	f	B	No symptom	AIDP	nr	nr	Yes	No	Partial	Italy
66	f	A	30	AIDP	nr	DM, AHT, arthritis	Yes	No	Partial	Iran
55	f	A	31	AMSAN	nr	COPD	Yes	Yes	Death	Iran
14	f	A	nr	nr	nr	No	Yes	No	Yes	Iran
38	m	A	16	AIDP	nr	No	PE	No	Partial	Iran
20-63	7 m	nr	nr	AIDP	nr	nr	Yes	No	Partial	UK, *n* = 7
65	m	A	5	AIDP	nr	DM, AHT	Yes	Yes	Death	Sudan
43	m	A	10	AIDP	nr	nr	Yes	No	Partial	Spain
63	m	A	1	MFS	nr	nr	No	No	Partial	UK
61	m	A	nr	MFS	No	nr	Yes	No	Yes	Germany
58	m	B	nr	AIDP	nr	nr	Yes	Yes	Partial	UK
70	f	A	15	AMAN	nr	AHTt, obesity	Yes, PE	No	Partial	Italy

A, onset of GBS after onset of non-neurological manifestations; *AAR* Aortic aneurysm repair; *AHT* Arterial hypertension; *AL* Alcoholism, *ASPC* Antibodies for SARS-CoV-2 positive in CSF; *AV* Artificial ventilation; B, onset of GBS before onset of non-neurological manifestations; *CHD* Coronary heart disease; *CIC* CoV2 in CSF; *CM* Comorbidities; *DM* Diabetes; *f* Female; *HLP* Hyperlipidaemia; *LC* Lung cancer; *LOO* Latency between onset of GBS and COVID-19 respectively vice versa; *m* Male; *nd* Not done; *nr* Not reported; *NCS* Nerve conduction study; *NTX* Renal transplantation; *pc* Personal communication; *PCB* Pharyngeal, cervical, brachial variant of GBS; *PE* Plasma exchange; *PNC* Polyneuritis cranialis; *RA* Rheumatoid arthritis; *RI* Renal insufficiency; *RSD* Reflex sympathetic dystrophy; *S* Steroids

**Table 2:** Summary of findings in 220 patients with SC2-GBS

Number of papers retrieved: 95	
Number of SC2-GBS: 220	
Number of patients with SC2-GBS subtypes: AIDP (*n* = 118), AMAN (*n* = 18), AMSAN (*n* = 11), MFS (*n* = 7), PNC (*n* = 2), PCB (*n* = 1), BSE (*n* = 0)	
Age range of patients: 8 to 94 years	
Gender: male (*n* = 146), female (67)	
Onset: after COVID-19 (*n* = 156), together with COVID-19 (*n* = 3), before COVID-19 (*n* = 6)	
Latency between onset of COVID-19 and GBS: − 10 to + 90 days	
Therapy: IVIG (*n* = 191), plasmapheresis (*n* = 15), steroids (*n* = 2), MV (*n* = 41)	
Outcome: CR (*n* = 37), PR (*n* = 119), death (*n* = 12)	

*CR* Complete recovery; *MV* Mechanical ventilation; *PR* Partial recovery

## Discussion

This systematic review shows that SC2-GBS is not due to a direct attack of the virus but rather due to an immunological reaction to the virus. It also shows that the number of reports about SC2-GBS is increasing and that the outcome is worse compared to non-SC2-GBS [6].

Though the number of cases with SC2-GBS is increasing suggesting that the overall prevalence of GBS has increased since the outbreak of the pandemic, there are conflicting results concerning this matter. In a UK study of 47 SC2-GBS patients, the prevalence of GBS did not increase between March 2020 and May 2020 as compared to the years 2016–2019 [6]. On the contrary, a retrospective, multi-centre study from northern Italy of 34 SC2-GBS patients showed that the estimated incidence of GBS in March 2020 and April 2020 increased from 0.93/100000/year in 2019 to 2.43/100000/year in 2020 [7]. There are several reasons why SC2-GBS may be missed and why the prevalence of GBS in fact increased since onset of the pandemic. First, SC2-GBS may go undetected due to misinterpretation as increased weakness or sensory disturbances of a pre-existing neuropathy. Second, SC2-GBS may be misinterpreted as critical ill neuropathy. Third, work-up for neuropathy may be incomplete due to mild manifestations or due to occurrence during ICU stay.

Before diagnosing SC2-GBS, it is crucial to exclude various differential diagnoses. These include previously existing neuropathy, critical ill myopathy, critical ill neuropathy, toxic neuropathy, or neuropathy or myopathy due to side effects of applied drugs. Lopinavir and tocilizumab have been reported to cause neuropathy [8, 9]. There are also reports indicating that chloroquine may induce neuropathy [10].

If GBS develops during immobilisation for artificial ventilation, diagnosing SC2-GBS becomes challenging [7]. In patients under artificial ventilation for COVID-19, SC2-GBS should be considered if clinical neurologic exam suggests neuropathy and if patients cannot be weaned from the respirator. In this case, nerve conduction studies and investigations of the CSF should be initiated. Diagnosing SC2-GBS is crucial as appropriate treatment may improve the overall outcome of COVID-19 patients [11].

In some cases, SC2-GBS develops before classical clinical manifestations of the infection [12] being explained by subclinical infection with the virus prior to onset of GBS or the incubation time of SARS-CoV-2, which is up to 14 days [7].

Though there are no prediction models for the outcome or the need of artificial ventilation in SC2-GBS patients available, there are indications that the outcome is poor if there are complications from hypercoagulability (stroke, pulmonary embolism) and if there are superinfections or sepsis.

Most of the studies included in this review did not specify if respiratory failure in SC2-GBS patients resulted from brainstem encephalitis, BFE, involvement of the respiratory muscles in GBS, from pneumonia ending up as acute, respiratory distress syndrome (ARDS), from pulmonary embolism, heart failure, or from mixtures of these conditions. Specifying the cause of respiratory failure however is crucial as treatment and outcome may differ significantly among these conditions.

## Conclusions

SC2-GBS is most likely secondary to an immune reaction against SARS-CoV-2 since the virus has not been found in CSF of any SC2-GBS patient reported. SC2-GBS occurs at any age. SC2-GBS does not differ from non-SC2-GBS regarding clinical presentation and treatment, but the outcome of SC2-GBS is worse compared to non-CS2-GBS patients. The prevalence/incidence of GBS most likely increased since the outbreak of the pandemic. Since there are no studies about the optimal treatment of SC2-GBS subtypes available, they should be treated empirically in the same way as non-SC2-GBS subtypes. Early diagnosis of SC2-GBS is warranted because if appropriate treatment is applied in due time, the overall outcome from the infection may improve.

## Data Availability

Not applicable

## Abbreviations

AIDP: Acute, inflammatory, demyelinating polyneuropathy
AMAN: Acute, motor axonal neuropathy
AMSAN: Acute, motor and sensory, axonal neuropathy
ARDS: Acute, respiratory distress syndrome
BFE: Bickerstaff encephalitis
CNS: Central nervous system
CSF: Cerebrospinal fluid
GBS: Guillain-Barre syndrome
IVIG: Intravenous immunoglobulins
MFS: Miller-Fisher syndrome
PCB: Pharyngeal, cervical, and brachial variant
PNC: Polyneuritis cranialis
PNS: Peripheral nervous system
SC2-GBS: SARS-CoV-2-associated GBS
